# Supplementary material for: Isolation and Bioassay of Linear Veraguamides from a Marine Cyanobacterium (Okeania sp.)
Source: Molecules. 2025 Feb 4;30(3):680. doi: 10.3390/molecules30030680 (PMC11819893; doi:10.3390/molecules30030680)

Supplemental Information

Isolation of Linear Veraguamides from a Marine Cyanobacterium (*Okeania sp.*)

Stacy-Ann J. Parker[1], Andrea Hough[1], Thomas Wright[1], Neil Lax[2], Asef Faruk[1], Christian K. Fofie[3], Rebekah Simcik[3], Jane E. Cavanaugh[1], Benedict J. Kolber[3], and Kevin J. Tidgewell[*1,4]

Table of Contents:

S 1: 1H-NMR spectrum of natural product **1** in CD_3CN at 500 MHz. pg 5

S 2: COSY-NMR spectrum of natural product **1** in CD_3CN at 500 MHz. pg 6

S 3: TOCSY-NMR spectrum of natural product **1** in CD_3CN at 500 MHz. pg 7

S 4: HSQC-NMR spectrum of natural product **1** in CD_3CN at 500 MHz. pg 8

S 5: HMBC-NMR spectrum of natural product **1** in CD_3CN at 500 MHz. pg 9

S 6: HRES-Mass spectrum of natural product **1** (positive ionization mode). pg 10

S 7: 1H-NMR spectrum of natural product **2** in CD_3CN at 500 MHz. pg 11

S 8: COSY-NMR spectrum of natural product **2** in CD_3CN at 500 MHz. pg 12

S 9: HSQC-NMR spectrum of natural product **2** in CD_3CN at 500 MHz. pg 13

S 10: HMBC-NMR spectrum of natural product **2** in CD_3CN at 500 MHz. pg 14

S 11: HRES-Mass spectrum of natural product **2** (positive ionization mode). pg 15

S12: 1H-NMR spectrum of natural 2 in CD_3OD at 500 MHz pg 16

S13: 1H-NMR spectrum of natural 3 in $CDCl_3$ at 500 MHz pg 17

Figure **S1**. ^{1}H NMR spectrum of compound (**1**) in CD_3CN at 500 MHz.

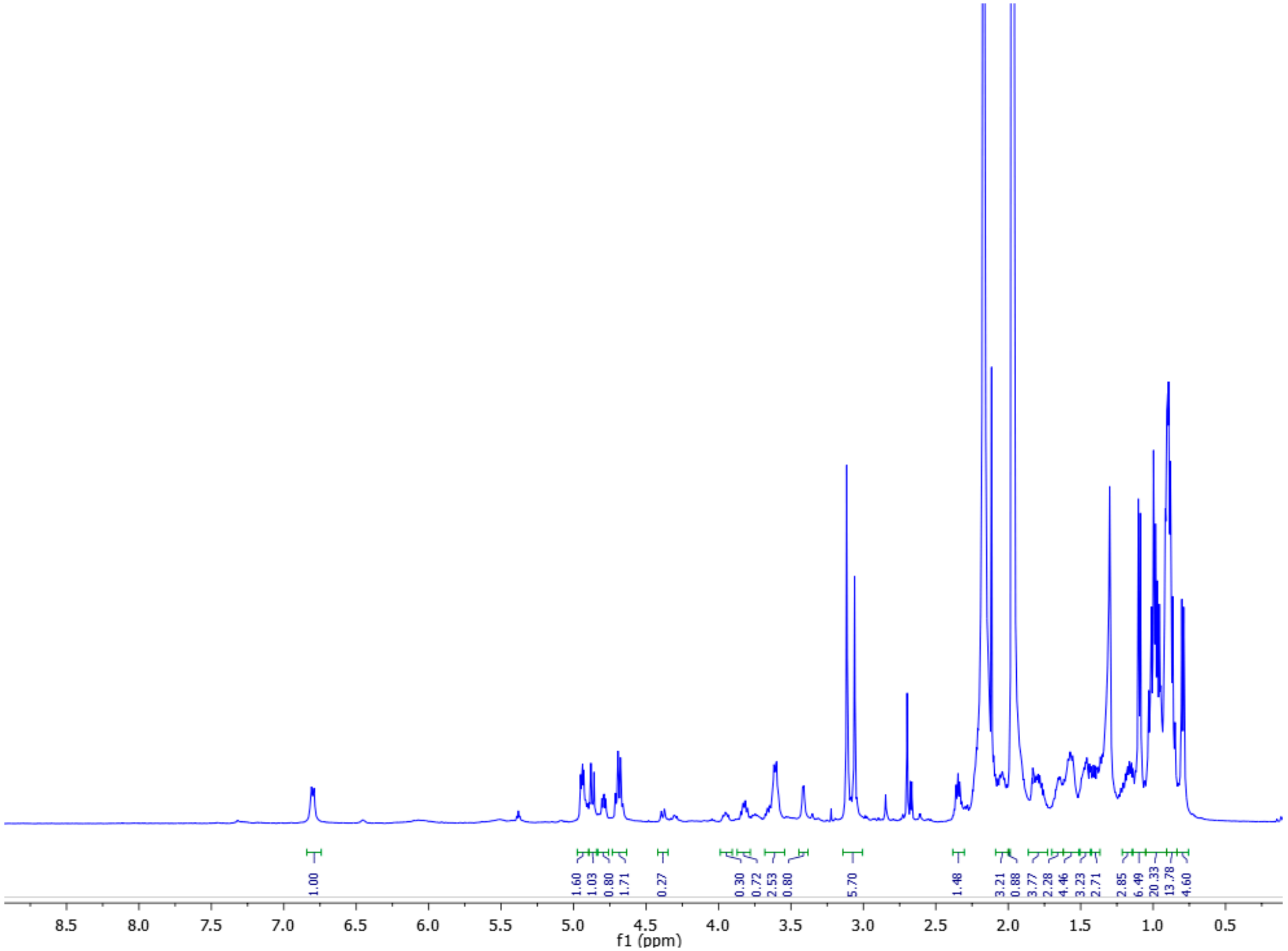

Figure S2. COSY spectrum of compound (**1**) in CD_3CN at 500 MHz.

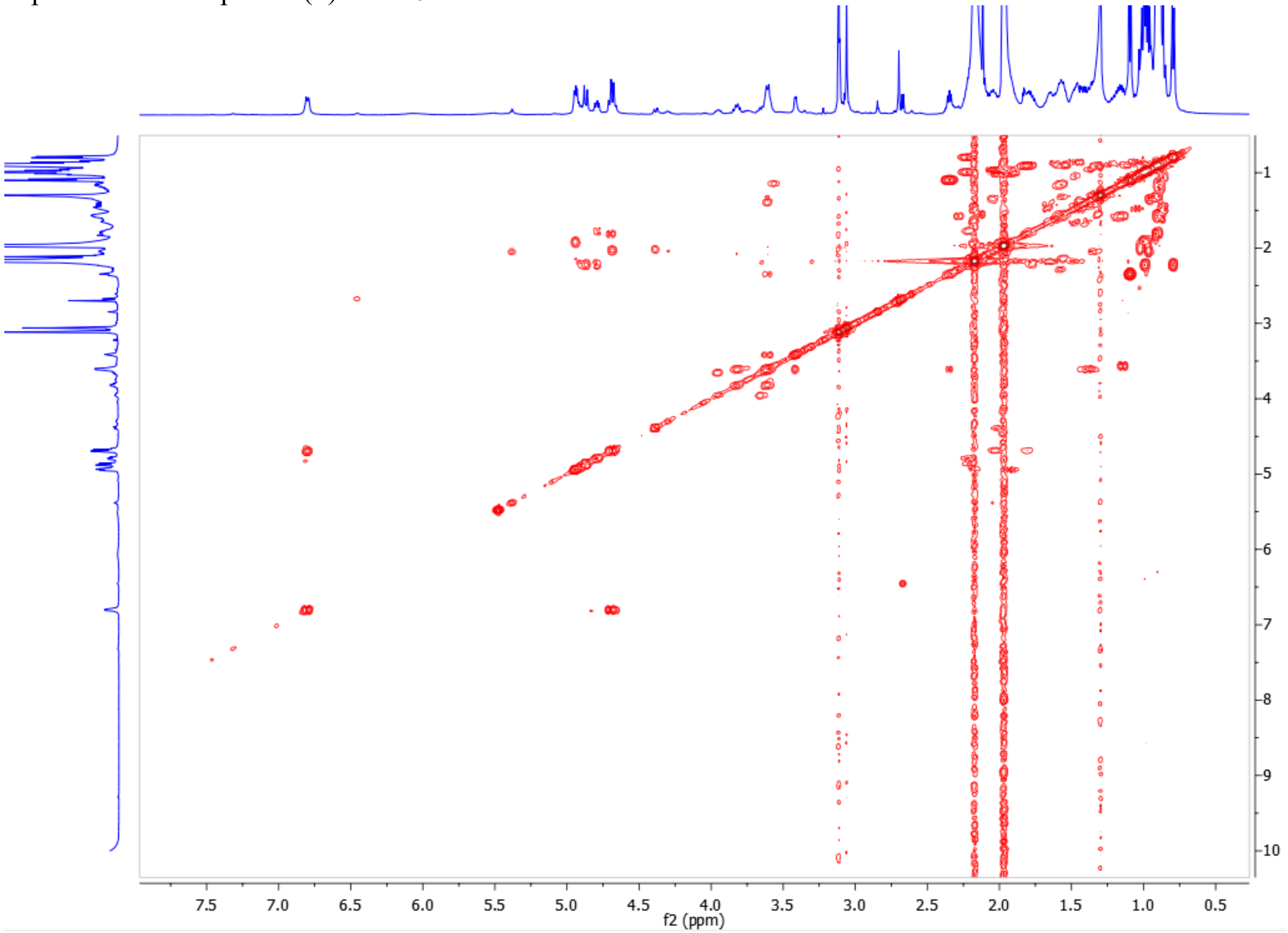

Figure S3. TOCSY spectrum of compound (**1**) in CD_3CN at 500 MHz.

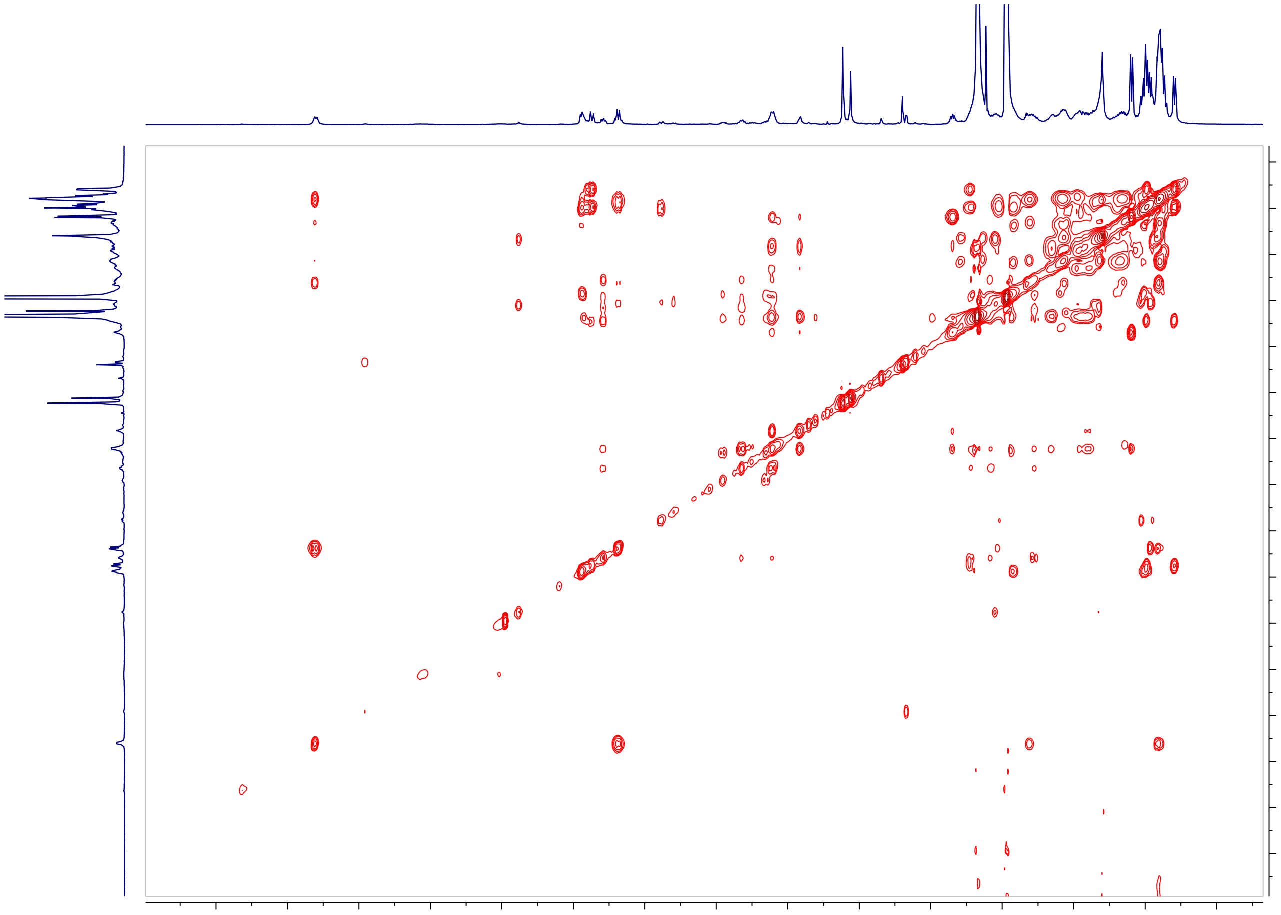

Figure S4. HSQC spectrum of compound (**1**) in CD_3CN at 500 MHz.

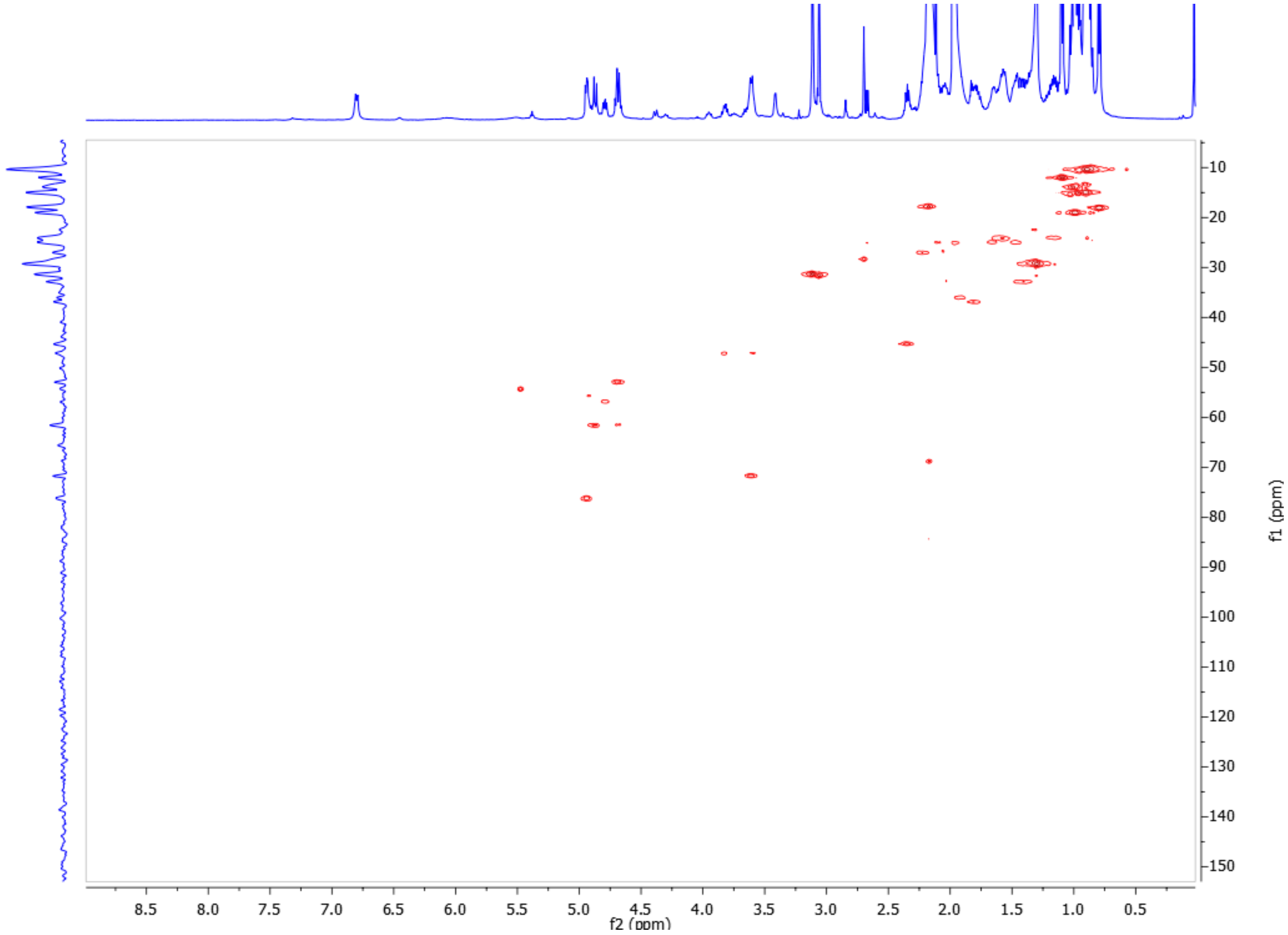

Figure S5. HMBC spectrum of compound (**1**) in $CDCl_3$ at 500 MHz.

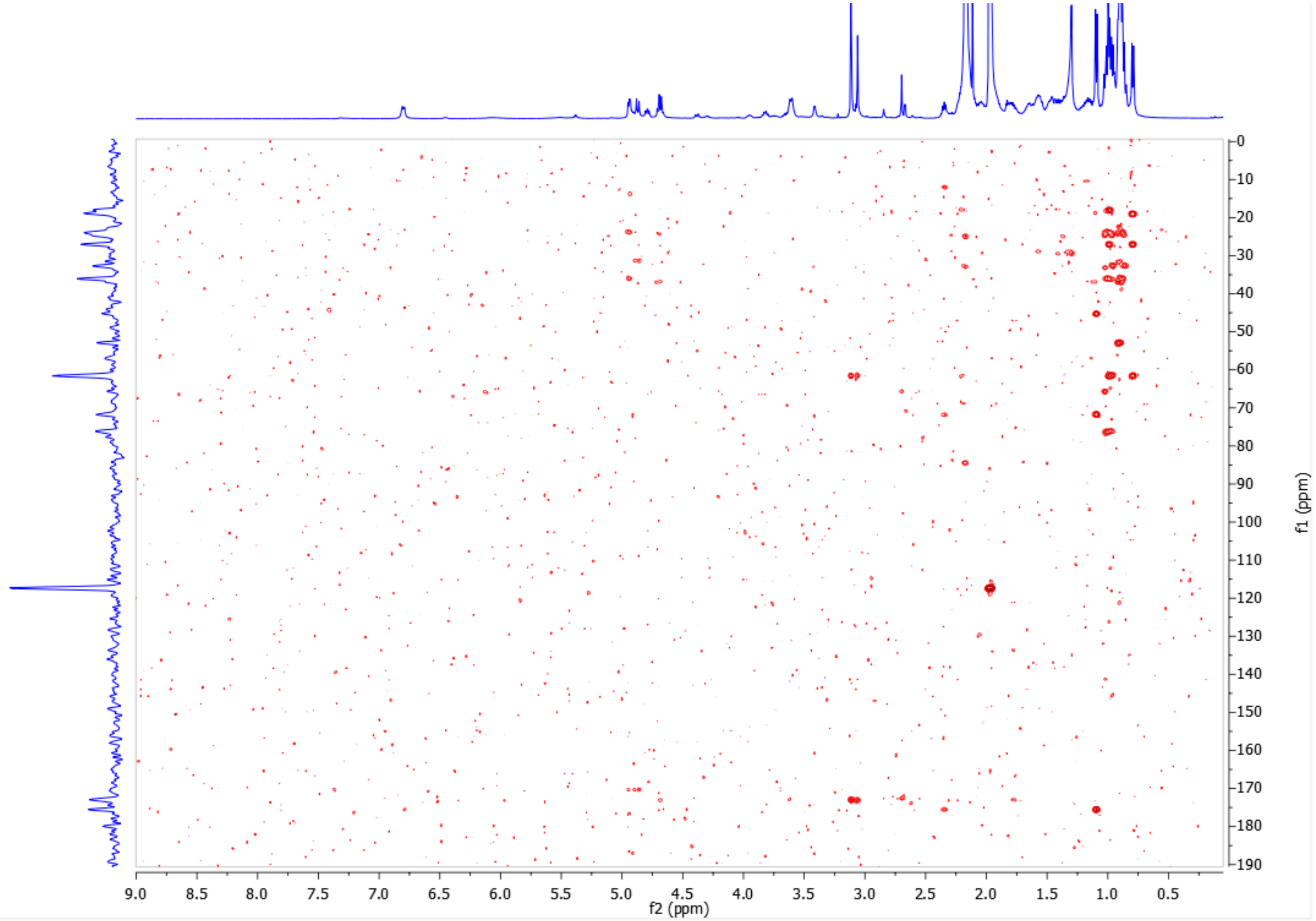

Figure S6. HRESIMS spectrum of (**1**).

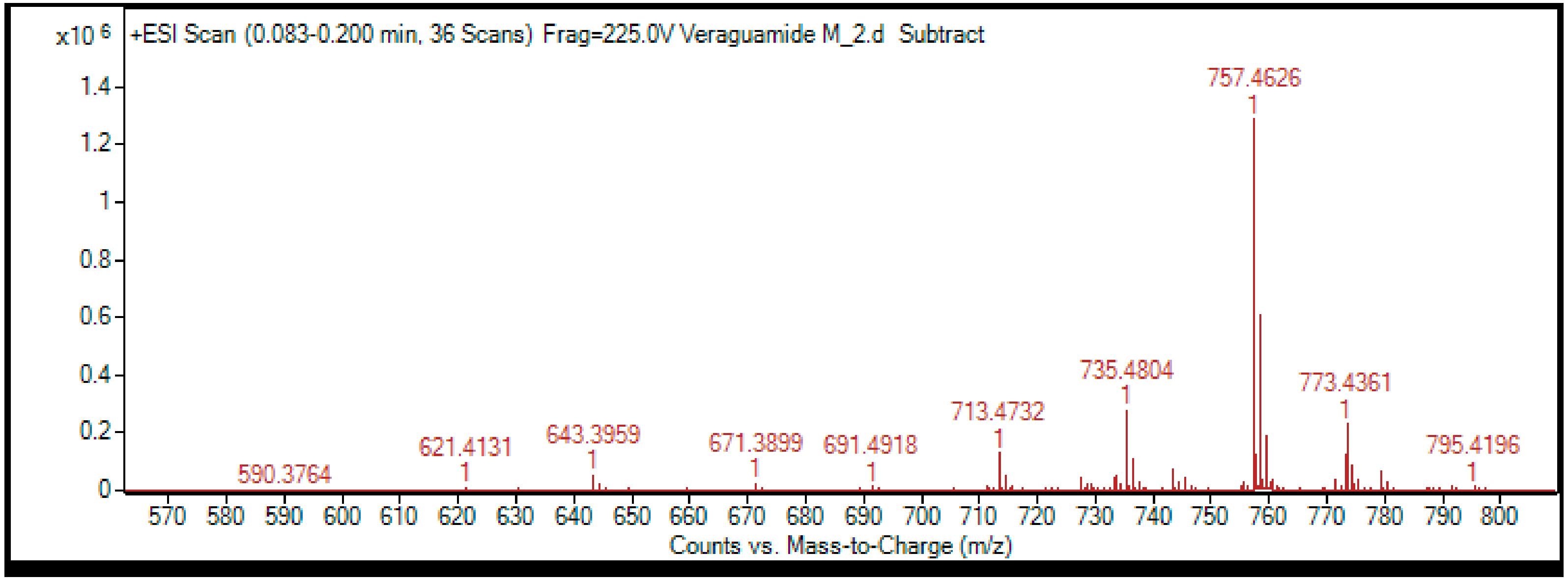

S 7: ^{1}H-NMR spectrum of *natural product* **2** in CD_3CN at 500 MH

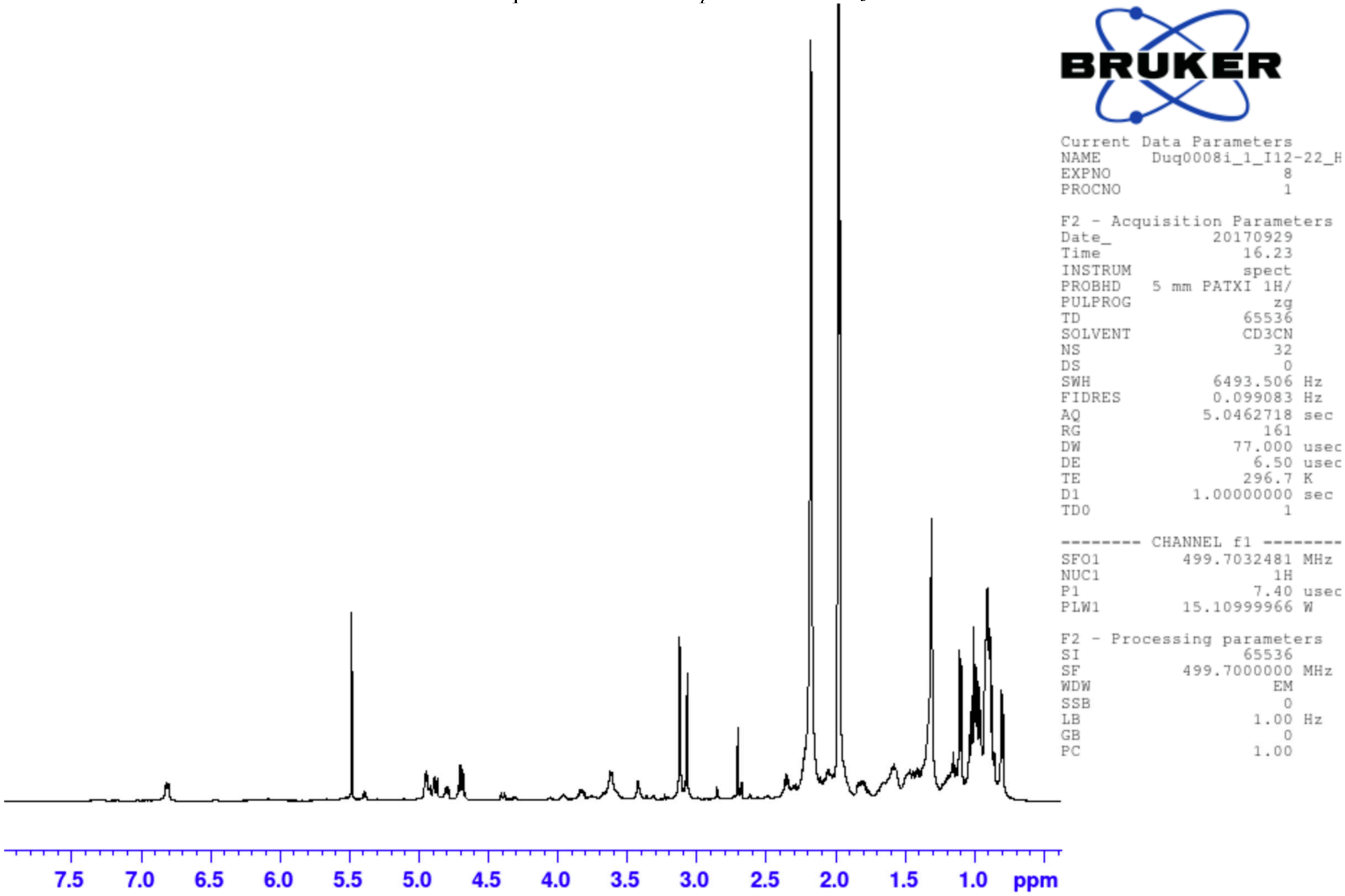

S 8: COSY-NMR spectrum of *natural product* **2** in CD_3CN at 500 MHz.

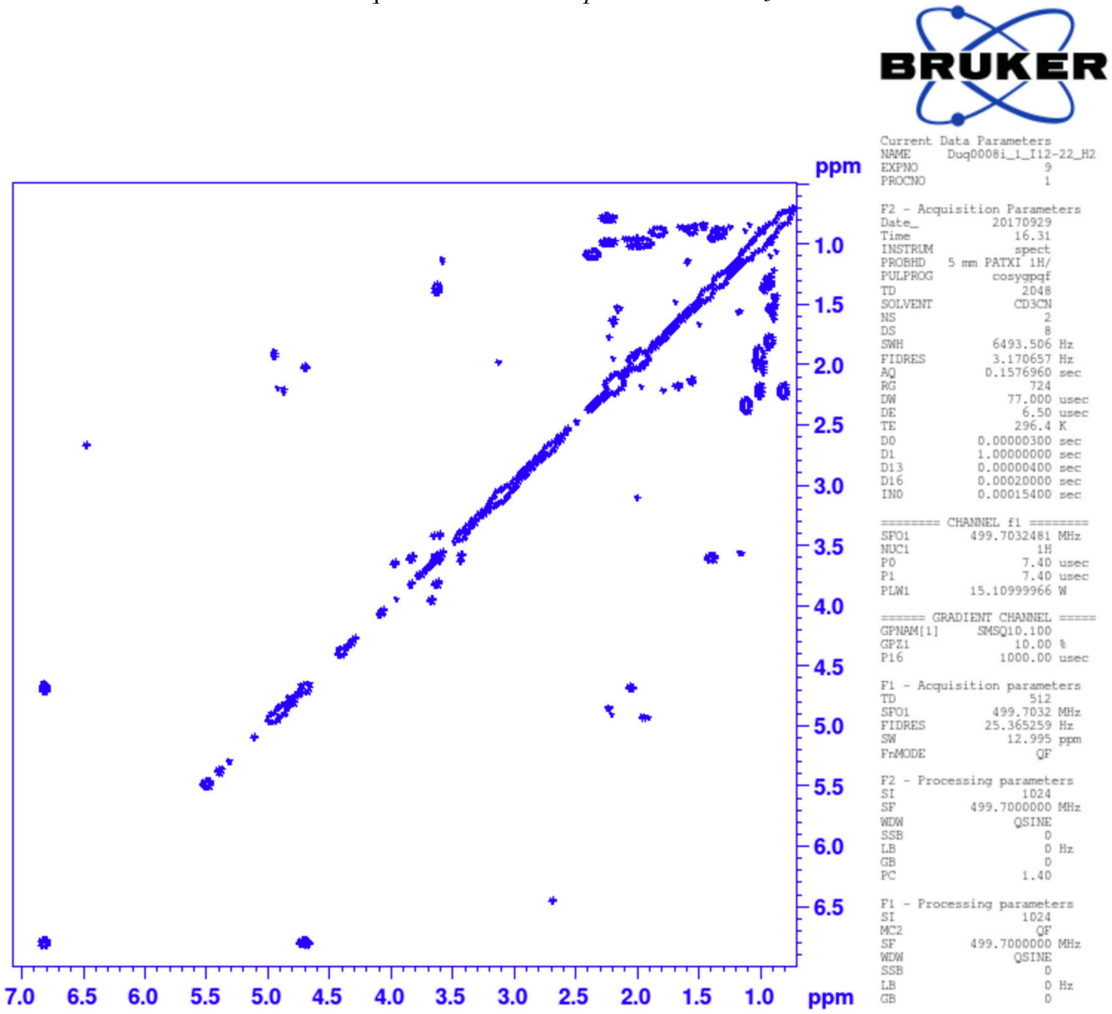

S 9: HSQC-NMR spectrum of *natural product* **2** in CD_3CN at 500 MHz.

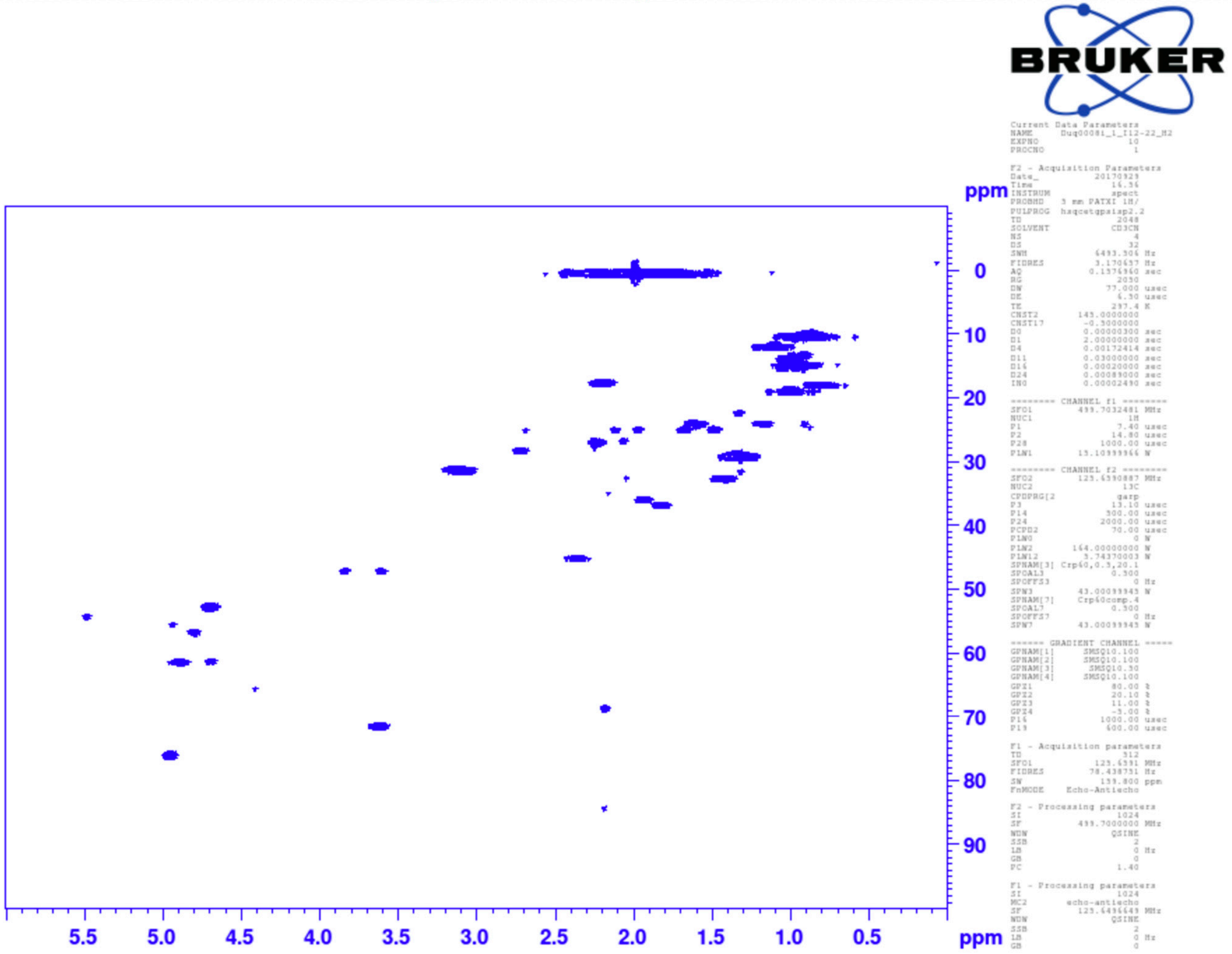

S 10: HMBC-NMR spectrum of *natural product* **2** in CD_3CN at 500 MHz.

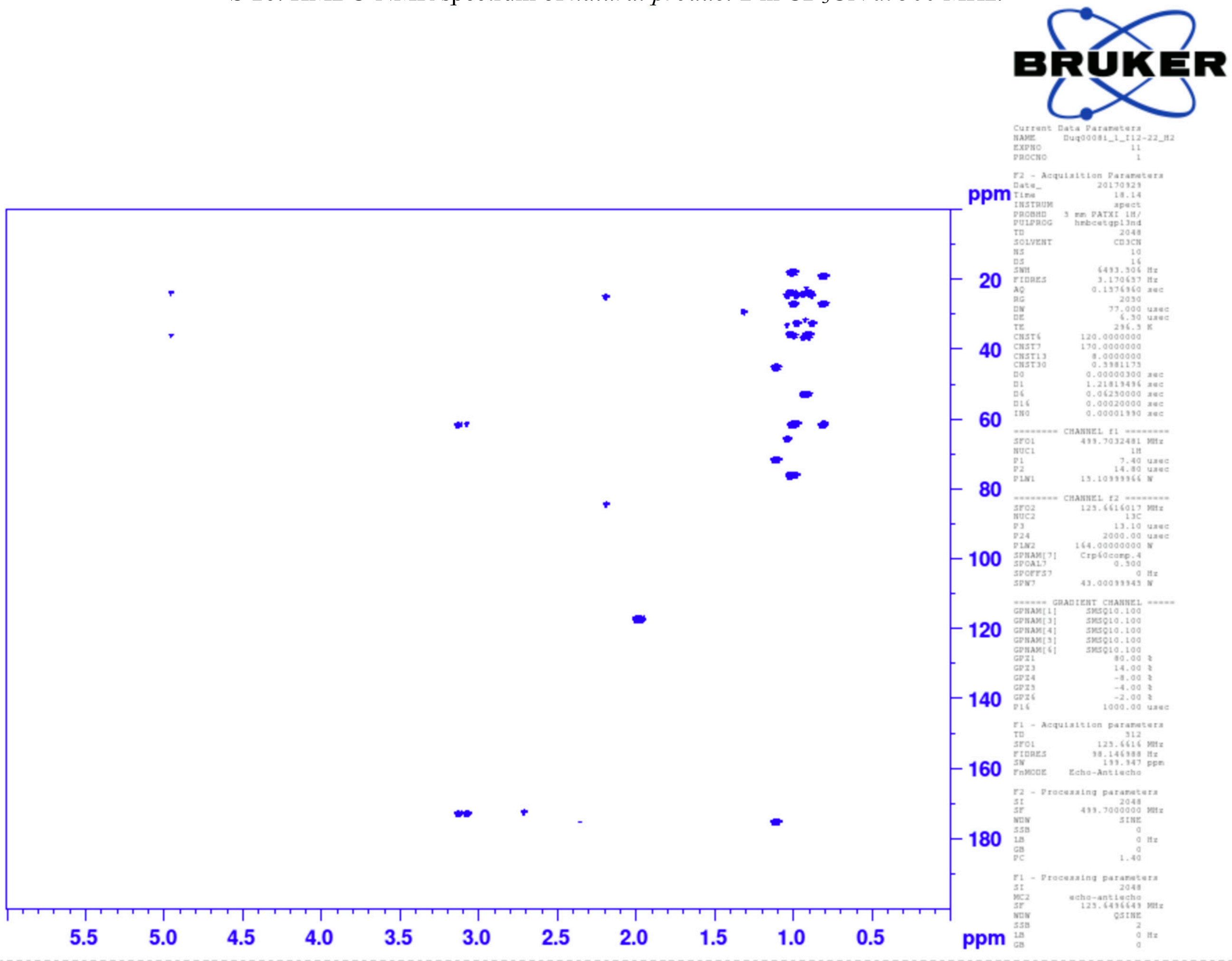

S 11: HRES-Mass spectrum of *natural product* **2** (positive ionization mode).

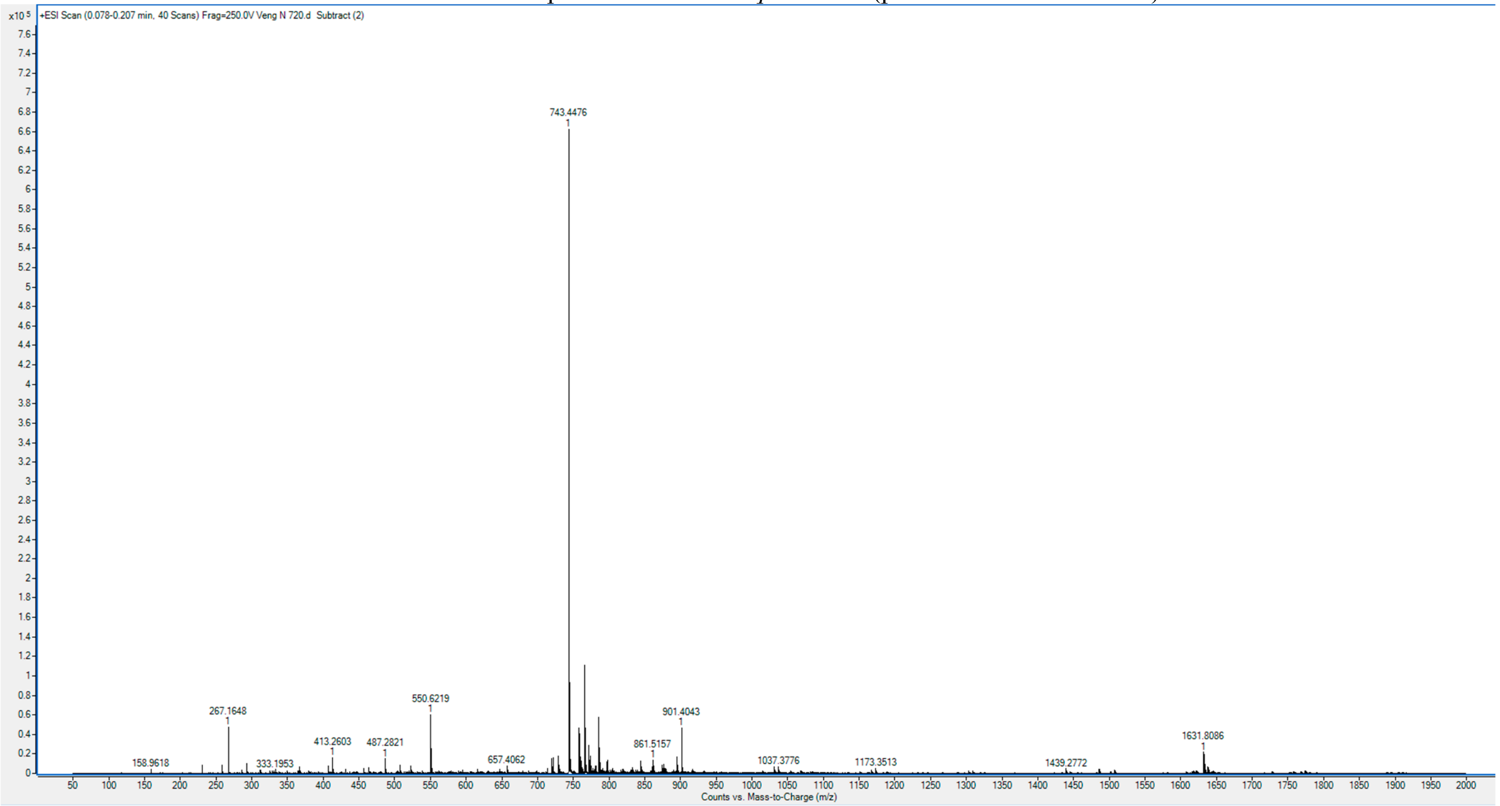

S 12: ^{1}H-NMR spectrum of *natural product* **2** in CD_3OD at 500 MHz.

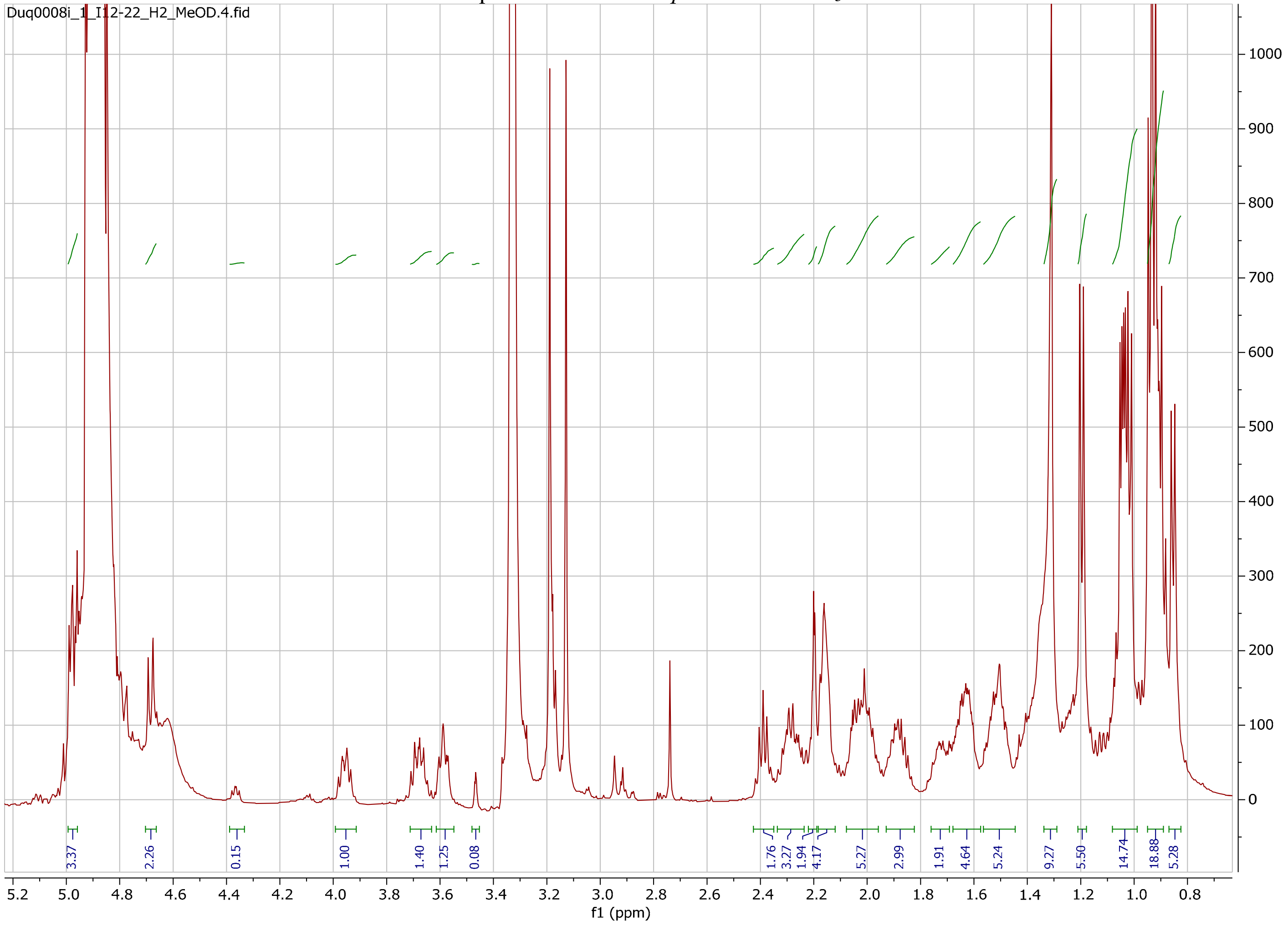

S13: 1H-NMR spectrum of natural product 3 in $CDCl_3$ at 500 MHz.

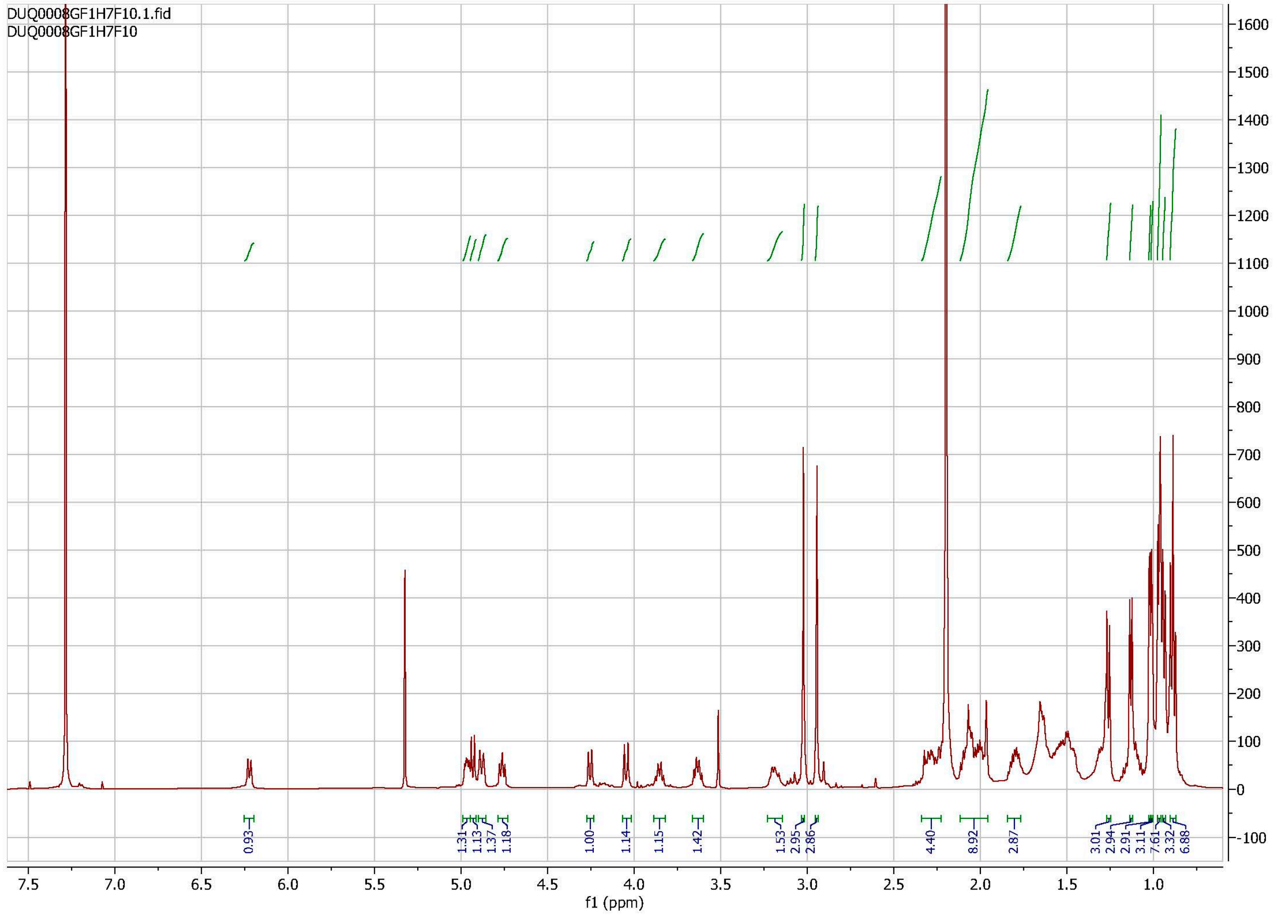

Supplement: Supplementary file 1 [file molecules-30-00680-s001.zip › molecules-3392336-supplementary.pdf]
